# Increased F_2_-Isoprostane Levels in Semen and Immunolocalization of the 8-Iso Prostaglandin F_2*α*_ in Spermatozoa from Infertile Patients with Varicocele

**DOI:** 10.1155/2018/7508014

**Published:** 2018-02-26

**Authors:** Giulia Collodel, Elena Moretti, Mariangela Longini, Nicola Antonio Pascarelli, Cinzia Signorini

**Affiliations:** ^1^Department of Molecular and Developmental Medicine, University of Siena, 53100 Siena, Italy; ^2^Rheumatology Unit, Azienda Ospedaliera Universitaria Senese, Policlinico Le Scotte, 53100 Siena, Italy

## Abstract

Polyunsaturated fatty acid damages lead to alterations in sperm function. This study aimed to investigate the involvement of F_2_-isoprostanes (F_2_-IsoPs), oxidized lipid products from arachidonic acid, in sperm quality impairment. For this purpose, F_2_-IsoP levels in semen and F_2_-IsoP localization in spermatozoa were explored in infertile subjects affected by idiopathic infertility or varicocele, as well as in fertile men. As compared to fertile men, in the idiopathic infertility and varicocele groups, sperm concentration, motility, morphology, viability, and fertility index were significantly lower and the mean scores concerning sperm apoptosis, necrosis, and immaturity were significantly higher. The idiopathic infertile group showed a reduction in sperm motility and fertility index, as well as an increase of apoptosis and necrosis percentages, in comparison to the varicocele group. The varicocele group showed the highest levels of F_2_-IsoPs, a significant increase of sperm immaturity, and a significant correlation between F_2_-IsoP levels and sperm immaturity. 8-Iso Prostaglandin F_2*α*_, biomarker of *in vivo* F_2_-IsoP, was clearly localized in sperm midpiece and cytoplasmic residues. Data show that F_2_-IsoP formation is relevant in semen and sperm from infertile patients with varicocele and high percentage of immaturity, suggesting that a correct fatty acid integrity is needed for sperm maturation.

## 1. Introduction

Sperm membrane is a key structure influencing sperm function; it is involved not only in sperm motility and vitality but also in acrosomal reaction and sperm-oocyte fusion. The characteristics and performance of the plasma membrane are strongly determined by its own fatty acid (FA) profile [1].

To this regard, mammalian spermatozoa are characterized by a high proportion of polyunsaturated fatty acids (PUFAs); in particular, testicular cells and spermatozoa contain large amounts of 20 and 22 carbon n-3 and n-6 PUFAs [2], which are considered as major constituents in human spermatozoa phospholipids [3–5]. Actually, the FA ratio appears to be critical to infertility in asthenozoospermic males [6], and FA composition has been suggested as a predictor of cryopreservation success of a seminal sample [7]. On this point, PUFAs were generally found higher in samples from normozoospermic men as compared to infertile patients [8–10] even if Khosrowbeygi and Zarghami [11] found higher levels of linoleic, arachidonic, and docosahexaenoic acids (DHA) in spermatozoa of patients with altered semen parameters compared to normozoospermic men. Moreover, Martinez-Soto et al. [7] demonstrated that spermatozoa the n-6/n-3 PUFA ratio was at a lower level in fertile men compared to the infertile ones, due to a significantly higher amount of total n-3 PUFA.

Oxidative stress (OS) results from a number of endogenous and exogenous stressors and is believed to play a central role in the pathogenesis of male infertility; when excessive amounts of reactive oxygen species (ROS) are produced or when antioxidant activity fails, the status of OS rises. It has been known that PUFAs represent the main target of the free radical insult, leading to the oxidative lipid deterioration causing alterations in sperm functional characteristics [12, 13].

To this regard, F_2_-isoprostanes (F_2_-IsoPs) are the most proximal products of the free radical-catalyzed arachidonic acid oxidation and are one of the most reliable approaches to evaluate a condition of endogenous lipid peroxidation *in vivo* [14–16]. Moreover, F_2_-IsoPs are not mere markers of OS, but they also elicit a wide variety of responses in different cell types [16, 17]. Initially formed in situ on phospholipids by esterified arachidonic acid peroxidation, F_2_-IsoPs are subsequently released in a free form in biological fluids [18]. Structurally similar to the prostaglandin F_2*α*_, (PGF_2*α*_), a total of 64 different isomers can be generated by peroxidation of the precursor arachidonic acid, being the 15-F_2t_-IsoP isomer (also referred to as 8-iso-PGF_2*α*_) currently used as biomarker of *in vivo* F_2_-IsoP formation [16].

Seminal plasma levels of catalase were significantly lower in infertile patients while the levels of free 8-iso-PGF_2*α*_ were significantly higher in infertile patients compared with normozoospermic men [11]. Moreover, significant negative correlations were found between malondialdehyde content and seminal superoxide dismutase activity and arachidonic acid content of spermatozoa from normozoospermic samples [19].

Varicocele has been associated with reduced male reproductive potential. Current evidence suggests the central role of ROS and the resultant OS in the pathogenesis of varicocele-associated male subfertility although the mechanisms have not yet been fully described [20] and it is likely to be multifactorial [21, 22] showing an alterated FA profile in a group of patients with varicocele compared with a group of normozoospermic men. In particular, a consistent reduction of DHA levels was found in infertile patients with varicocele compared to fertile men [23]. Although it has been reported that OS is a key element in the pathophysiology of varicocele-related infertility [24], no information on the kind of PUFA involved has been reported.

The purpose of this study was to assess the role of arachidonic acid peroxidation in infertile patients with varicocele by investigating F_2_-IsoP levels in semen and 8-iso-PGF_2*α*_ localization in sperm. A comparison with fertile men and subjects with idiopathic infertility was carried out.

## 2. Materials and Methods

### 2.1. Patients

Selected semen samples were obtained from 38 patients (aged 26–40 years) attending our centre for semen analysis. All patients were infertile; they were not able to get their partners pregnant after 2 years of unprotected sexual intercourses. Twenty-five patients showed the presence of varicocele detected by physical examination and scrotal Eco-color Doppler. Thirteen patients had an idiopathic infertility. All patients satisfied these inclusion criteria: nonazoospermic patients with a normal 46,XY karyotype evaluated by conventional cytogenetic analysis; a normal hormone profile; no history of radiotherapy, chemotherapy, chronic illness, or medication; patients without sperm defects of possible genetic origin; and absence of clinically asymptomatic genitourinary infections and leukocytospermia.

A control group consisted of 13 men with proven fertility. Fertile men (aged 27–39 years) showed a normal karyotype and semen parameters > 25 percentile, [25]; they were not affected by anatomical problems and/or infections. These fertile men fathered one child during the past 3 years.

Eligible patients and controls did not take FA supplementation in the previous one year. All patients and controls gave informed consent for this research.

### 2.2. Semen Analysis

Participants were instructed to abstain from intercourse and masturbation for 3–5 days before the sperm collection. Semen samples were collected by masturbation in a sterile container and were examined after liquefaction for 30 min at 37°C. Volume, pH, concentration, motility, and morphology (Papanicolaou (PAP) test modified for spermatozoa) were assessed as recommended by WHO 2010 [25]. Peroxidase stain was used for leukocyte identification; a value of >1 × 10^6^ leukocytes/mL was considered out of range [25]. Sperm vitality was assessed by staining 10 *μ*L of the semen samples with 10 *μ*L of 0.5% eosin Y (CI 45380) in 0.9% aqueous sodium chloride solution. A few minutes after staining, the samples were observed by light microscope and stained (dead) cells and unstained (living) cells were scored.

After analysis, the samples were centrifuged at 2000 rpm for 15 min; the seminal plasma was stored at −80°C until use; butylated hydroxytoluene (BHT) (90 *μ*M) was added to the seminal plasma as an antioxidant.

### 2.3. TEM Analysis

For the TEM procedures, sperm samples were fixed in a cold Karnovsky fixative and maintained at 4°C for 2 h. Then the semen was washed in 0.1 mol/L cacodylate buffer (pH 7.2) for 12 h, postfixed in 1% buffered osmium tetroxide for 1 h at 4°C, and washed again in 0.1 mol/L cacodylate buffer; the samples were dehydrated in a graded ethanol series and embedded in Epon Araldite. Ultrathin sections were cut with a Supernova ultramicrotome (Reickert Jung, Vienna, Austria), mounted on copper grids, stained with uranyl acetate and lead citrate, and then observed and photographed with a Philips CM12 transmission electron microscope (TEM; Philips Scientifics, Eindhoven, the Netherlands, Centro di Microscopie Elettroniche “Laura Bonzi,” ICCOM, Consiglio Nazionale delle Ricerche (CNR),Via Madonna del Piano 10, Firenze, Italy).

Three hundred sperm sections were analyzed from each sample. Two highly trained examiners (E.M., G.C.), blind to the experiment, used the same evaluation criteria to estimate and record the main submicroscopic characteristics. The TEM data was elaborated using a mathematical formula [26] which provides numerical scores such as fertility index (number of sperm free of structural defects in a semen sample) and percentage of sperm pathologies such as immaturity, apoptosis, and necrosis [27], defined by distinctive ultrastructural characteristics. The typical traits of sperm immaturity include the presence of cytoplasmic droplets, altered acrosomes, and roundish or elliptical nuclei with uncondensed chromatin. Marginated chromatin, translucent vacuoles included in cytoplasmic residues, and swollen and badly assembled mitochondria are the ultrastructural indicators of apoptosis. Whereas, sperm with reacted or absent acrosome misshaped nuclei with disrupted chromatin and broken plasma membrane and poor axonemal and periaxonemal cytoskeletal structures are affected by necrosis [28].

### 2.4. F_2_-IsoP Determination

For total (sum of free and esterified) F_2_-IsoP determination in the seminal plasma, samples were stored at −80°C until assay. At the time of the assays, each sample was incubated (45°C for 45 min) in the presence of aqueous 1M KOH (500 *μ*L/mL). Subsequently, the pH was adjusted to 3.0 by adding 1 M HCl (500 *μ*L/mL). Each sample was spiked with tetradeuterated derivative of PGF_2*α*_ (PGF_2*α*_-d_4_) (500 pg), as an internal standard. Subsequently, each sample was applied to an octadecylsilane (C_18_) cartridge followed by an aminopropyl (NH_2_) cartridge and isoprostanes were eluted. After that, the F_2_-IsoP carboxylic group was derivatized as the pentafluorobenzyl ester whereas the hydroxyl groups were converted to trimethylsilyl ethers. Finally, F_2_-IsoP determinations were carried out by gas chromatography/negative ion chemical ionization tandem mass spectrometry (GC/NICI-MS/MS) analysis. The measured ions were *m/z* 299 and *m/z* 303 derived from the [M-181]^−^ precursor ions (*m/z* 569 and *m/z* 573) produced from the derivatized 15-F_2t_-IsoP (i.e., 8-iso-PGF_2*α*_, the most represented isomer for F_2_-IsoP measurement) and PGF_2*α*_-d_4_, respectively [29, 30].

### 2.5. Immunocytochemistry

Sperm samples were washed in phosphate buffer saline, smeared on glass slides, air dried, and processed as previously reported [31]. Briefly, the slides were incubated overnight at 4°C with rabbit polyclonal anti-8-iso-PGF_2*α*_ antibody (Abcam, Cambridge, UK), diluted at 1 : 100; reaction was revealed by an anti-rabbit FITC conjugate antibody raised in a goat (Sigma-Aldrich, Italy), diluted at 1 : 300. Incubation in the primary antibodies was omitted in the control samples. Slides were mounted with 4,6-diamidino-2-phenylindole (DAPI) solution (Vysis, Downers Grove, IL). Observations were made with a Leica DMI 6000 Fluorescence Microscope (Leica Microsystems, Germany), and the images were acquired by the Leica AF6500 Integrated System for Imaging and Analysis (Leica Microsystems, Germany). At least five hundred sperm from each sample was examined.

### 2.6. Statistical Analysis

The variables (sperm concentration, progressive motility, morphology, vitality, fertility index, apoptosis, necrosis, and immaturity) among the three considered groups (fertile men, idiopathic infertility, and varicocele) were analyzed by the Kruskal-Wallis test. When a statistically significant difference was found among groups, the Mann–Whitney *U* test was used between pairs of groups. In the table, all the variables are expressed as means, medians, and SD. The Spearman's rank correlation coefficient (rho) was used to discover the strength of a relationship between the investigated variables. *p* < 0.05 was considered statistically significant. Statistical analysis was performed with the Package R (Version 3.3.1) Project management with RStudio (Version 0.99.1292 - (c) 2009-2016 RStudio Inc.).

## 3. Results

In the selected men, semen volume and pH, sperm concentration, progressive motility, morphology, and vitality were evaluated according to WHO guidelines [25] and analyzed by light microscopy. Sperm ultrastructure was detected by TEM analysis mathematically elaborated. TEM values related to the fertility index and the percentage of sperm pathologies [27]. In the seminal plasma of the examined individuals, F_2_-IsoPs were also quantified by using the GC/NICI-MS/MS analysis.

Men were divided into three groups: fertile, men with idiopathic infertility, and infertile with varicocele, and data are reported in Table 1. Fertile men were used as control. All analyzed variables, except volume and pH, were significantly different in the three groups (*p* < 0.01; Table 1).

Sperm concentration, motility, morphology, and vitality were significantly (*p* < 0.01) lower in the idiopathic infertile and infertile varicocele groups as compared to fertile men. The means of these variables in the idiopathic infertile group were significantly reduced compared to the infertile varicocele group, with the single exception of not significant sperm motility's reduction.

Fertile men showed a significant increase in fertility index (*p* < 0.01) compared to the other examined groups, and this variable was higher also in the varicocele group in respect to the idiopathic infertile group. The mean scores concerning sperm apoptosis, necrosis, and immaturity were significantly reduced in fertile men. In the idiopathic infertile group, the percentages of apoptosis and necrosis were significantly higher (*p* < 0.01) in comparison with those observed in the varicocele group. In the latter, immaturity percentage was significantly increased compared to those detected in fertile men and the idiopathic infertility group (*p* < 0.01).

F_2_-IsoP level percentage increased significantly in the infertile varicocele group in comparison with fertile men and the idiopathic infertility group (*p* < 0.01). F_2_-IsoP levels did not differ between fertile men and the idiopathic infertility group.

In Table 2, the correlations among the variables of this study calculated in each different selected group were reported; we particularly focused on the relationship between F_2_-IsoP levels and semen variables. F_2_-IsoP levels positively correlated with immaturity in the varicocele group (*p* < 0.05). Moreover, a positive correlation between sperm motility and fertility index and sperm morphology was found in fertile men. In the idiopathic group, sperm concentration positively correlated with sperm morphology, vitality, and fertility index and negatively with necrosis; sperm morphology negatively correlated with necrosis and positively with vitality. Necrosis negatively correlated with sperm motility, vitality, and fertility index.

Finally, in the varicocele group, necrosis negatively correlated with sperm morphology, vitality, and fertility index; also, apoptosis negatively correlated with vitality (*p* < 0.05).

**Figure 1:**
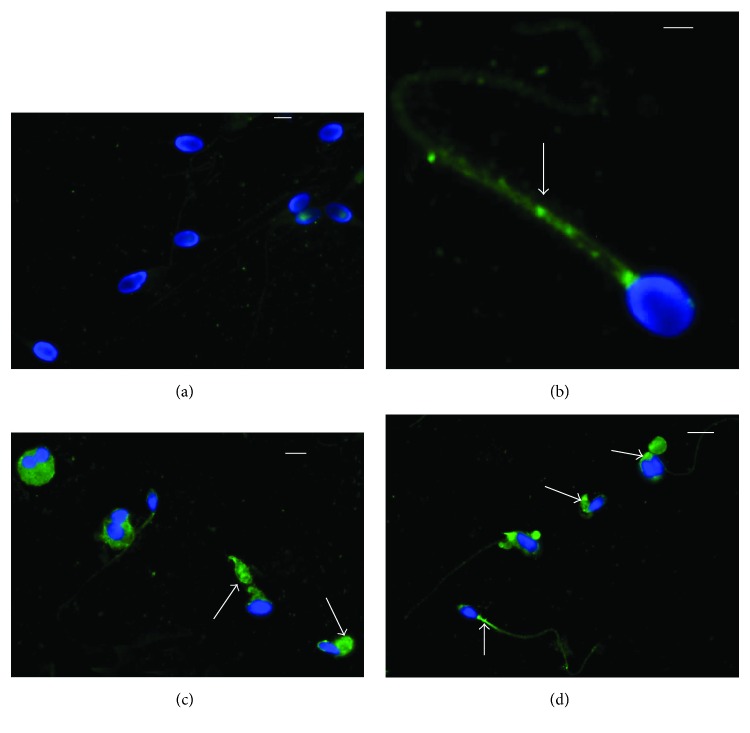
Immunocytochemical staining with polyclonal 8-iso-PGF_2*α*_ antibody of sperm from fertile man (a), idiopathic infertile man (b), infertile patients with varicocele (c, d). In the sperm from a fertile man, the signal is absent; in the sperm from an idiopathic infertile man, the label is dotted (arrow) and located in the midpiece of the sperm tail. A high percentage of spermatozoa from infertile patients with varicocele shows an intense staining in the cytoplasmic residues surrounding the head and the tail and in the midpiece (arrows). Bar: 5 *μ*m.

**Figure 2:**
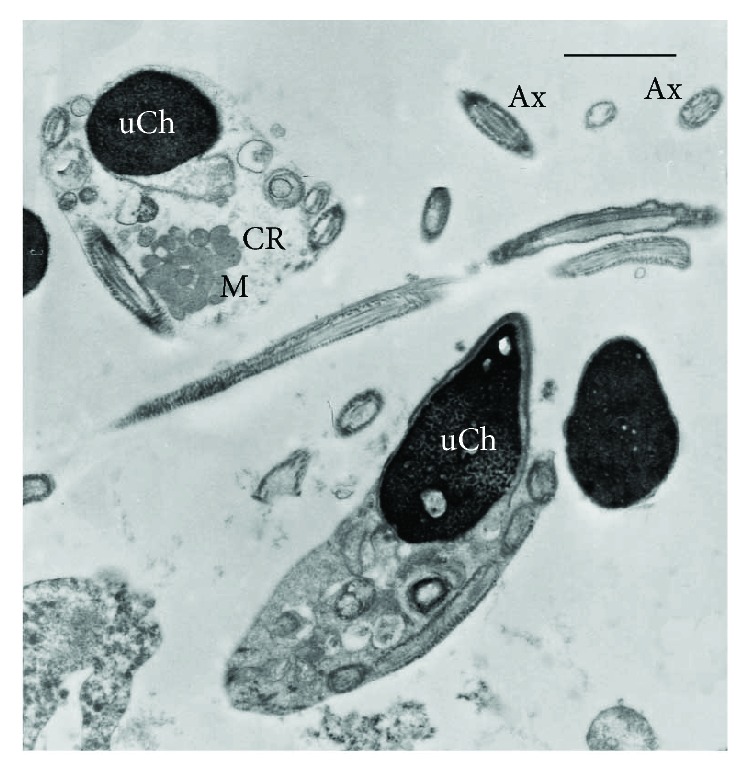
TEM micrograph of longitudinal and cross sections of immature sperm characterized by irregular nuclei with uncondensed chromatin (uCh). Cytoplasmic residues (CR) are present embedding swollen mitochondria (M) and coiled disassembled axonemes (Ax). Bar: 1 *μ*m.

Immunofluorescence showed the presence of 8-iso-PGF_2*α*_ in sperm cell whose localization resulted variable in patients with different seminal conditions. In spermatozoa from fertile men, the labelling was almost absent (90–95%; Figure 1(a)); in patients with idiopathic infertility, the label was absent in about 65% of spermatozoa; when it was present, the signal was weakly localized and dotted in the neck region and along the tail (Figure 1(b)). In spermatozoa from infertile patients affected by varicocele, immunofluorescence staining of 8-iso-PGF_2*α*_ was present in almost 68% of spermatozoa; it appeared to be particularly abundant in spermatozoa with characteristics of immaturity. The sperm alterations characterizing this pathology are clearly described by TEM (Figure 2): uncondensed chromatin, the presence of cytoplasmic residues and coiled tails. The label was clearly localized in the cytoplasm surrounding the sperm head and coiled tails, sometimes in the tail at the mitochondrial level (Figures 1(c) and 1(d)).

## 4. Discussion

In the present study, we investigated F_2_-IsoP formation in semen and spermatozoa from infertile subjects affected by varicocele, as well as from men with idiopathic infertility and fertile men. The F_2_-IsoP levels were correlated with sperm parameters and sperm TEM indices. We found that in infertile varicocele patients, the higher levels of semen 8-iso-PGF_2*α*_ were related to the higher percentage of sperm immaturity. The localization of 8-iso-PGF_2*α*_ in spermatozoa were detected by immunofluorescence.

As F_2_-IsoPs originate from the arachidonic acid free radical-induced oxidation, our study falls in the issues related to the relevance of both FA profile and OS in the semen quality.

The importance of lipid composition, in particular phospholipids in the plasma membrane and semen plasma, for spermatozoa function has since long been recognized [32]. The FA profile has received attention both in relation to sperm fertility and for nutritional implications, and it has been proposed as a marker of semen quality for patients with different semen parameters [22]. Likewise, OS has been implicated in male infertility also considering oxidative lipid damage. In particular, free radicals, and specifically ROS, are able to attack polyunsaturated FAs in cell membranes, thus generating the prostaglandin-like end product IsoPs. As FAs are a relevant energy source, their oxidative alteration can lead to a decrease of spermatozoa efficiency. The relevance of a correct FA composition in semen was reinforced in light of the significant decreased sperm motility after incubation of sperm with a FA oxidation inhibitor [33].

Varicocele is the most common risk factor for male infertility; however, not all males with varicocele experience infertility, since some patients with varicocele have normal spermatogenesis. The mechanisms of varicocele-associated infertility are not completely understood. An increase of ROS has been associated with male fertility complications including varicocele and idiopathic infertility [34]. Mathematically elaborated TEM analysis is a tool able to quantify, for each semen sample, the percentage of sperm pathologies [26]. In sperm from patients with varicocele, an increase of sperm immaturity may be detected [35, 36]. Sperm immaturity is a pathology characterized by peculiar ultrastructural alterations such as round- or elliptical-shaped nuclei, uncondensed chromatin, and the presence of double-nucleus sperm, coiled tail, and cytoplasmic residues.

In this paper, the infertile varicocele group, despite having a similar low sperm quality with idiopathic infertile patients compared with fertile men, interestingly, have increased levels of F_2_-IsoPs and percentage of sperm immaturity; in addition, the higher levels of semen 8-iso-PGF_2*α*_ significantly correlated with sperm immaturity.

Immunofluorescence investigation of 8-iso-PGF_2*α*_ indicated that the majority of sperm from fertile men did not show the labeling; on the contrary, the 35% of spermatozoa from infertile idiopathic patients displayed a weak, dotted staining located in the sperm tail. In infertile patients with varicocele, the label was observed in a high percentage of sperm in cytoplasmic residues, which are abundant around the sperm heads surrounding the coiled tail. Moreover, the signal was clearly detected in the midpiece of the sperm tail. The localization in the sperm tail of 8-iso-PGF_2*α*_ in the group of patients with idiopathic infertility and varicocele and the absence in the sperm tail of fertile men seem to suggest the presence of an oxidative damage that could be partially related to the reduced sperm motility. Zerbinati et al. [22] analyzed a group of patients with varicocele showing a reduced number of sperm and motility and observed an altered FA profile compared with the normozoospermic group.

However, our results seem to also indicate a role of cytoplasmic residue in the increased presence of F_2_-IsoPs in the varicocele group, also considering that both free and esterified F_2_-IsoP forms were evaluated at the same time in semen samples.

A cytoplasmic droplet is referred as abnormal when it is >1/3 to 1/2 the size of the sperm head [25]. Cytoplasmic droplets less than that size, present as vesicles at the neck of the sperm, are likely normal remnants of cytoplasm on sperm produced by a normal testicle. Studies suggest that small droplets are normal structures of spermatozoa [37], whereas large cytoplasmic droplets are related to a variety of pathologies, as well as varicocele [38]. Many studies in several animal species investigated the role of cytoplasmic droplets abundant in epididymal sperm [39]; sperm collected from cauda epididymis are used in reproductive techniques, but the role of sperm droplets in mammalian sperm, whether ejaculated or epididymal, is still a matter of debate. These cytoplasmic residuals may increase ROS production, and their antioxidant enzyme activity may play an important role during the sperm maturation process, as reported in dogs [40]. On these bases, the hypothesis on the relationship between F_2_-IsoPs and sperm immaturity which arises from our data appears to be reinforced.

Human spermatozoa are different because the cytoplasmic droplet remains at the neck region. In this paper, the abnormal cytoplasmic residues seem to be attacked by OS as well as the mitochondrial helix located in the middle piece of the sperm tail where in infertile patients with varicocele, a clear signal was detected. It is known that in men, excess residual cytoplasm harbours ROS that can damage sperm membrane lipids, proteins, and DNA and can disrupt sperm function [41]; however, recently, a successful pregnancy in a couple with severe male factor infertility after selection of sperm with small cytoplasmic droplets was reported [42]. The increased levels of F_2_-IsoPs in semen from infertile patients with varicocele seem to be due only partially to alteration of sperm parameters such as concentrations, progressive motility, vitality, or sperm pathologies such as necrosis and apoptosis since they are highlighted also in idiopathic infertile men but mostly connected to the presence of sperm immaturity. The measurement of F_2_-IsoPs may have a prognostic value in those diseases in which a role for OS has been implicated and may then indicate that a correct fatty acid integrity is needed for sperm maturation.

The possibility of antioxidant supplementation has been considered in treatment of male infertility [43]. Dietary FA influence on sperm FA profiles has been reported in different studies [3, 44–46], and it seems that sperm FA profiles are most sensitive to dietary omega-3 PUFA [5].

To this regard, recently Kolahdooz et al. [47] suggested that the beneficial effect of consumption of 5 mL *N. sativa* oil daily on sperm quality of infertile men may be related to the chemical FA contents.

A consistent reduction of DHA levels was found by Tang et al. [23] in infertile men with varicocele. DHA levels appear to be critical in FA oxidation occurrence, as investigated in erythrocytes which are considered as a model for cell membranes [48]. To this regard, it has been shown that an unfavorable saturated FA/PUFA ratio in the red blood cell membrane from patients with Rett syndrome is associated to an increased F_2_-IsoP plasma levels, which are normalized after a n-3 supplementation. Thus, the increased availability in DHA appears to be implicated in reducing the arachidonic acid oxidation [49].

## 5. Conclusion

A deeper knowledge on sperm ultrastructure and immunocytochemistry as well as the FA oxidative status could be of help in sperm immaturity diagnosis and could indicate a therapeutic FA-personalized dietary integration.

## Figures and Tables

**Table 1:** Semen parameters (means ± standard error and medians) of men classified into three groups (fertile controls, men with idiopathic infertility, and infertile men with varicocele).

Variables	Diagnosis			Statistics	
	Fertile men	Idiopathic infertile	Infertile varicocele	Kruskal-Wallis	Mann–Whitney *U* test
*Sperm/mL*				*p* < 0.01	^∗∗^Fertile versus idiopathic; ^∗∗^fertile versus varicocele; ^∗∗^idiopathic versus varicocele
Media ± SD	146.73 ± 65.55	10.82 ± 16.63	68.96 ± 65.94		
Median	153.00	1.70	49.00		

*Mot* (%)				*p* < 0.01	^∗∗^Fertile versus idiopathic; ^∗∗^fertile versus varicocele
Media ± SD	53.38 ± 18.81	30.31 ± 28	33.08 ± 25.2		
Median	52.00	22.00	31.00		

*Morphology* (%)				*p* < 0.01	^∗∗^Fertile versus idiopathic; ^∗∗^fertile versus varicocele; ^∗^idiopathic versus varicocele
Media ± SD	18.31 ± 8.28	6.77 ± 4.59	10.68 ± 5.29		
Median	16.00	9.00	11.00		

*Vitality* (%)				*p* < 0.01	^∗∗^Fertile versus idiopathic; ^∗∗^fertile versus varicocele; ^∗^idiopathic versus varicocele
Media ± SD	82.77 ± 6.46	60.15 ± 21.38	73.48 ± 11.11		
Median	85.00	60.00	75.00		

*FI*				*p* < 0.01	^∗∗^Fertile versus idiopathic; ^∗∗^fertile versus varicocele; ^∗^idiopathic versus varicocele
Media ± SD	7471850.69 ± 8904134.66	230072.23 ± 428981.78	794594.64 ± 721287.10		
Median	3804591.00	17484.00	674322.00		

*A* (%)				*p* < 0.01	^∗∗^Fertile versus idiopathic; ^∗∗^idiopathic versus varicocele
Media ± SD	4.07 ± 1.81	11.84 ± 7.61	4.29 ± 1.08		
Median	4.06	9.32	4.13		

*N* (%)				*p* < 0.01	^∗∗^Fertile versus idiopathic; ^∗∗^idiopathic versus Varicocele
Media ± SD	30.46 ± 6.86	47.10 ± 17.34	31.43 ± 9.35		
Median	29.05	42.84	32.88		

*I* (%)				*p* < 0.01	^∗∗^Fertile versus idiopathic; ^∗∗^fertile versus varicocele; ^∗∗^idiopathic versus varicocele
Media ± SD	42.63 ± 9.68	53.61 ± 6.27	71.45 ± 8.27		
Median	44.91	54.00	73.58		

*F*_2_ *-IsoPs (ng/mL)*				*p* < 0.01	^∗∗^Fertile versus varicocele; ^∗∗^idiopathic versus varicocele
Media ± SD	17.22 ± 12.08	24.30 ± 50.40	85.19 ± 44.27		
Median	13.33	7.20	88.06		

vol: volume in mL; sperm/mL: the number of spermatozoa/mL; Mot (%): the percentage of sperm with rapid + slow progressive motility; morphology (%): the percentage of sperm with normal morphology; vitality (%): the percentage of viable sperm evaluated by optical microscopy; FI: fertility index is a number indicating the fertility power of ejaculated sperm—the number of sperm probably is devoid of defects; *A* (%): the percentage of sperm apoptosis; *N* (%): the percentage of sperm necrosis; *I* (%): the percentage of sperm immaturity evaluated by TEM; F_2_-IsoPs: semen F_2_-IsoP levels evaluated by the GC/NICI-MS/MS analysis (ng/mL). ^∗^*p* < 0.05; ^∗∗^*p* < 0.01.

**Table 2:** Correlations (rho Spearman's coefficient) among all variables given in columns in three considered groups.

Variables	Investigated groups	Sperm/mL	Mot (%)	Morphology (%)	Vitality (%)	FI	*A* (%)	*N* (%)	*I* (%)
Mot (%)	Fertile men	NS							
	Idiopathic infertile	NS							
	Infertile varicocele	NS							

Morphology (%)	Fertile men	NS	*0.636* ^∗^						
	Idiopathic infertile	*0. 668* ^∗^	NS						
	Infertile varicocele	*0.425* ^∗^	*0.660* ^∗^						

Vitality (%)	Fertile men	NS	NS	NS					
	Idiopathic infertile	*0.554* ^∗^	NS	*0.510* ^∗^					
	Infertile varicocele	*0.469* ^∗^	*0.510* ^∗^	*0.680* ^∗^					

FI	Fertile men	NS	*0.600* ^∗^	*0.883^∗^*	NS				
	Idiopathic infertile	*0.861* ^∗^	NS	NS	NS				
	Infertile varicocele	*0.552* ^∗^	*0.536* ^∗^	NS	*0.571* ^∗^				

*A* (%)	Fertile men	NS	NS	NS	NS	NS			
	Idiopathic infertile	NS	NS	NS	NS	NS			
	Infertile varicocele	NS	NS	NS	*−0.504* ^∗^	NS			

*N* (%)	Fertile men	NS	NS	NS	NS	NS	NS		
	Idiopathic infertile	NS	−0.*619*^∗^	*−0.787* ^∗^	*−0.823* ^∗^	*−0.686* ^∗^	NS		
	Infertile varicocele	NS	*−0.609* ^∗^	*−0.423* ^∗^	*−0.657* ^∗^	*−0.559* ^∗^	NS		

*I* (%)	Fertile men	NS	NS	NS	NS	NS	NS	NS	
	Idiopathic infertile	NS	NS	NS	NS	NS	NS	NS	
	Infertile varicocele	NS	NS	NS	NS	NS	NS	NS	

F_2_-IsoPs (ng/mL)	Fertile men	NS	NS	NS	NS	NS	NS	NS	NS
	Idiopathic infertile	NS	NS	NS	NS	NS	NS	NS	NS
	Infertile varicocele	NS	NS	NS	NS	NS	NS	NS	*0.771* ^∗^

Sperm/mL: the number of spermatozoa/mL; Mot (%): the percentage of sperm with rapid + slow progressive motility; morphology (%): the percentage of sperm with normal morphology; vitality (%): the percentage of viable cells; FI: fertility index is a number indicating the fertility power of ejaculated sperm—the number of sperm probably is devoid of defects; *A* (%): the percentage of sperm apoptosis; *N* (%): the percentage of sperm necrosis; *I* (%): the percentage of sperm immaturity; F_2_-IsoP: semen F_2_-IsoP levels (ng/mL) evaluated by the GC/NICI-MS/MS analysis. *p* values are reported when significant: ^∗^*p* < 0.05.

## Abbreviations

F_2_-IsoPs: F_2_-isoprostanes
FA: Fatty acid
PUFAs: Polyunsaturated fatty acids
DHA: Docosahexaenoic acid
OS: Oxidative stress
ROS: Reactive oxygen species
PGF_2*α*_: Prostaglandin F_2*α*_
WHO: World Health Organization
BHT: Butylated hydroxytoluene
TEM: Transmission electron microscope
GC/NICI-MS/MS: Gas chromatography/negative ion chemical ionization tandem mass spectrometry
DAPI: 4,6-Diamidino-2-phenylindole.
